# Management of hot flashes in women with breast cancer

**DOI:** 10.3747/co.v17i1.473

**Published:** 2010-02

**Authors:** L. Kligman, J. Younus

**Keywords:** Breast cancer, hot flashes, tamoxifen, aromatase inhibitors

## Abstract

Hormone-suppression therapies are used for the treatment of breast cancer in the adjuvant and metastatic settings alike. However, side effects—including hot flashes—are frequently reported by patients as a cause of therapy discontinuation. This paper presents an overview of hormonal therapies and the evidence-based management options for hot flashes, summarized in a treatment algorithm.

## 1. INTRODUCTION

In 2008, an estimated 22,400 women in Canada were diagnosed with breast cancer, making that disease the most common cancer in women. Treatment includes any combination of surgery, chemotherapy, and radiation therapy. For women with cancers positive for the estrogen or the progesterone receptor (or both), hormone suppression is an additional option. Because hormone-suppressive treatment can span five or more years, care is often shared with or transferred to family physicians and community nurse practitioners.

In premenopausal women, the ovaries produce about 95% of circulating estrogen; in postmenopausal women, conversion or aromatization of androstenedione to estrone in peripheral tissues is the major source of estrogen. Approximately half of all women diagnosed with breast cancer will have a hormonesensitive tumour. Blocking uptake of estrogen at the receptor level or suppressing estrogen and progesterone production therefore becomes a goal of treatment.

## 2. OVERVIEW OF HORMONE THERAPIES

Tamoxifen, a selective estrogen receptor modulator, has both antagonist and agonist properties, blocking the effect of estrogen in breast tissue, but showing estrogen-like activity in other tissues. The indication for the use of tamoxifen has been expanded to include adjuvant treatment of both pre- and postmenopausal women. Male breast cancers commonly express the estrogen and progesterone receptors and can also be treated with tamoxifen.

Unlike tamoxifen, fulvestrant is a pure estrogen receptor antagonist with no agonist effects. It works by both downregulating and degrading the estrogen receptor. It is given once monthly as an injection. Currently, it is indicated for use only in hormone-sensitive metastatic breast cancer.

The identification of aromatase as an essential enzyme in the peripheral conversion of testosterone to estradiol led to the development of further options for hormone suppression. Aromatase inhibitors are used only in postmenopausal women to prevent stimulation of ovarian estrogen production through negative feedback to the hypothalamus and pituitary (analogous to the functional properties of fertility drugs). Current agents in this class include anastrozole, letrozole, and exemestane.

Hormone suppression is now standard treatment for pre- and postmenopausal women with advanced and early forms of hormone-sensitive breast cancer. Clinical trials such as atac (Anastrozole versus Tamoxifen Alone or in Combination), big (Breast International Group) 1-98, and ies (Intergroup Exemestane Study) have shown a small but significant additional benefit of aromatase inhibitors alone or in sequential combination with tamoxifen in improving disease-free survival^1^–^3^. Other trials are ongoing.

Almost all national and international guidelines recommend inclusion of an aromatase inhibitor in the treatment plan for hormone-positive tumours in postmenopausal women. However, for both pre- and postmenopausal women, frequently reported side effects include hot flashes, arthralgia, weight gain, hair thinning, dyspareunia, vaginal dryness, and loss of libido. In some cases, these side effects may be severe enough for patients to stop, independently reduce the dosage of, or otherwise alter potentially life-saving treatment regimens. Information from clinical trials has revealed that approximately 25% of women do not adhere to therapy and that this percentage increases to 50% for women not in clinical trials^4^. Because aromatase inhibition or tamoxifen treatment is intended to last for at least 5 years, successful management of these side effects becomes paramount in increasing patient compliance and optimal outcome. The present review focuses on management options for one of these side effects: hot flashes.

## 3. CLINICAL DESCRIPTION AND PATHOPHYSIOLOGY

Hot flashes are commonly defined as recurring transient episodes of flushing and sweating, with a sensation of heat, often accompanied by palpitations or anxiety, and sometimes followed by chills^5^. A population-based survey comparing women having breast cancer with women in the general population determined that breast cancer survivors were 5.3 times more likely than women in the general population to experience menopausal symptoms^6^.

Several theories have been posited for the pathophysiology of hot flashes. A decline in estrogen has been suggested to cause a change in the thermoregulatory set point in the anterior portion of the hypothalamus^7^. The thermoregulatory nucleus initiates perspiration and vasodilatation to keep core body temperature within a well-regulated range called the thermoregulatory zone^8^. Researchers have demonstrated a narrowing of the zone between sweating and shivering in symptomatic women, so that small elevations within the zone cause a change in hormones or neurotransmitters, producing a hot flash^9^. Yet correlations of the frequency and severity of hot flashes with plasma or serum estrogen levels are poor, suggesting that other mechanisms may be involved^7^.

Serotonin has repeatedly been proved to be an important regulator of body temperature, and its action appears to be time- and dose-dependent^10^. For example, drug-induced excess of 5-hydroxytryptamine has been noted to induce malignant hyperthermia^11^. The thermoregulatory set point has been suggested to possibly depend on a balance of two or more serotonin receptors^12^. Changes in the balance, possibly related to the direct or indirect action of estrogen, could theoretically induce a vasomotor response.

Functional estrogen receptors have been found in human vascular smooth muscle, suggesting that estrogen may also have a direct and important role in vascular function^13^. Recent research has also shown a correlation between plasma estradiol levels and baroreflex sensitivity^14^.

The regulation of core body temperature is likely to be ultimately found to involve complex interactions of norepinephrine, estrogen, testosterone, serotonin, and endorphins in neuroendocrine pathways.

Hot flashes emerged as a significant adverse effect in all of the aromatase inhibitor trials (Table I). The target trial (Trial to Assess Response to Gefitinib in EGFR-mutated Tumors) compared anastrozole with tamoxifen as first-line treatment in postmenopausal women with advanced breast cancer. Hot flashes were reported in 19.6% of women receiving anastrozole as compared with 18.8% of those receiving tamoxifen^15^. In the atac adjuvant trial comparing anastrozole with tamoxifen, 35.7% of women on anastrozole reported hot flashes as compared with 40.9% of women on tamoxifen (*p* < 0.0001)^16^. Letrozole and tamoxifen were compared in the big 1-98 trial, and hot flashes were reported in 33.5% of women receiving letrozole and in 38% of those receiving tamoxifen (*p* < 0.001)^a^. The ies trial compared the steroidal aromatase inhibitor exemestane with tamoxifen. In that trial, hot flashes were reported in 42% of patients treated with exemestane and in 39.6% of those receiving tamoxifen (*p* = 0.082)^3^.

Little information is available about the duration or severity of hot flashes, but the ies trial did publish quality- of-life data indicating that this symptom decreased over time^17^. Some predictors of increased hot flash severity or frequency are known. These include previous history of estrogen replacement therapy, history of moderate to severe hot flashes during menopause in postmenopausal women, premature treatment-induced menopause at a young age, obesity, and smoking (direct relationship with number of cigarettes smoked)^18^.

## 4. REVIEW OF MANAGEMENT OPTIONS

**FIGURE 1:**
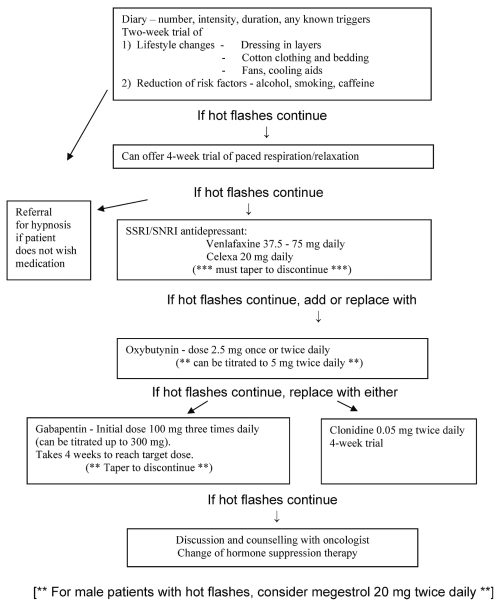
Treatment algorithm for hot flashes, based on the experience of the London Regional Cancer Program.

In 2004, a nurse-run hot flash clinic was started at the London Regional Cancer Program. The purpose of the clinic was to evaluate evidence-based treatment of hot flashes and to advance symptom management research. A stepwise approach based on efficacy data, tolerability, and local clinical experience is suggested for management (Figure 1, Table II).

Inclusion of a survey of side effects as part of the follow-up assessment of women receiving hormone-suppressive therapy is imperative. The initial assessment of hot flashes should include onset, baseline number and severity, effect on measures of quality of life such as sleep and work, and any known patterns or triggers. Often, patterns emerge and links can be made with triggers such as caffeine, smoking, and alcohol. Reduction of these risk factors is recommended. It is sometimes helpful to review lifestyle interventions such as dressing in layers, use of cotton clothing and bedding, strategic placement of electric fans, and the carrying of cold drinks. Reusable cooling bandanas containing polymer crystals may be helpful. They can be soaked in cold water for 10–30 minutes and tied around the neck for a long-lasting cooling effect. Results from these changes are usually seen within the first 1 to 2 weeks of use.

In small, controlled investigations, progressive muscle relaxation and paced respiration techniques have been shown to significantly reduce objectively measured hot flash occurrence^19^,^20^. In a larger randomized controlled trial of 150 women with primary breast cancer who were trained in deep-breathing techniques, muscle relaxation, and guided imagery, hot flash severity declined significantly over 1 month. Distress also significantly declined, but no difference in quality of life between the control group and the treatment group was observed^25^.

Hypnosis may also provide a non-pharmacologic option for women who prefer to avoid medications. In 2005, a case report of the successful use of hypnosis to treat hot flashes and rheumatic symptoms was published^27^. In a subsequent randomized trial of hypnosis as an intervention for hot flashes, a 68% reduction in hot flash scores was demonstrated in 60 breast cancer survivors as compared with a control group. Additional benefit was seen in parameters such as sleep, anxiety, and depression^24^.

Estrogen replacement, the most effective treatment for menopausal symptoms, is generally not recommended after a diagnosis of breast cancer. The habits (Hormonal Replacement Therapy After Breast Cancer Diagnosis—Is It Safe?) trial randomized 434 women with a previous diagnosis of breast cancer to receive either hormone replacement therapy (hrt) or best supportive care without hormones^28^,^29^. The endpoint of the trial was any new breast cancer event. After 2.1 years, 26 women in the hrt group (versus 8 in the no-hrt group) had experienced a new breast cancer event (relative hazard: 3.5; 95% confidence interval: 1.5 to 8.1). The trial was terminated December 17, 2003, because of unacceptable risk.

Breast cancer patients suffering hot flashes are often bombarded with information from well-intentioned friends and family members regarding alternative and complementary remedies, and those remedies are seen by many women with breast cancer as a more attractive option than traditional medicines. Unfortunately, few placebo-controlled trials of non-pharmacologic agents have been conducted, and those that have been conducted show marginal benefits at best^30^. Other studies are problematic because of small sample size and conflicting results. Most of these remedies are phytoestrogens that are structurally similar to estradiol. Phytoestrogens can be found in foods such as beans, seeds, and grains. They include products such as black cohosh, red clover, evening primrose oil, and soy, and depending on the dose, they work either to block estrogen receptors or to mimic estrogen. Their effect on an estrogen-sensitive breast cancer cell is unknown, and at the present time there is not enough evidence to advise women that these products are safe.

Clinical trials of pharmacologic agents have noted the significant placebo effect that any intervention can have on hot flashes. It has been reported in numerous clinical trials that the placebo effect can decrease hot flashes by approximately 50%^8^. Data collected from patient diaries and outcomes are typically reported in two ways: using a composite hot flash score consisting of the frequency, duration, and mean severity of each episode; or using separate effects, such as change in frequency and severity.

A few pharmacologic agents have shown varying degrees of efficacy for treating hot flashes. For some of these, side effects limit their usefulness; others show statistical benefit, but not clinically significant reductions in symptom frequency or intensity. The more successful agents include selective serotonin reuptake inhibitor (ssri) antidepressants, megestrol acetate, and gabapentin. Antihypertensives such as clonidine have shown modest benefit.

Clonidine, a centrally active alpha agonist that reduces vascular reactivity and norepinephrine release, is used principally for the management of hypertension. It is available in pills and transdermal patches. Transdermal clonidine was found to reduce the frequency of hot flashes by 20% and the severity by a modest 10%^31^. In another trial, Pandya *et al.* randomized 194 postmenopausal women on adjuvant tamoxifen therapy to receive either a placebo or oral clonidine 0.1 mg daily for 8 weeks. Decrease in frequency of hot flashes was greater for the clonidine group (38%) than for the placebo group (20%), with benefit also noted in measures of intensity and duration^32^. Other studies have shown increasing benefit with higher doses^33^.

The ssri antidepressants have also shown efficacy, with the greatest response noted in the breast cancer population. Loprinzi *et al.* conducted a randomized placebo-controlled trial of venlafaxine in 191 breast cancer survivors complaining of hot flashes^21^. Within that group, 69% were receiving tamoxifen. The trial randomized women to one of four treatment groups: three groups received venlafaxine in doses of 37.5 mg, 75 mg, and 150 mg; the fourth group received placebo. After 4 weeks, benefit was seen in the 75 mg dose group, with 63% of participants reporting more than 50% reduction in hot flashes. No additional benefit was seen by increasing the dose to 150 mg. A later study by Evans *et al.* confirmed these results in a non–breast cancer population^34^. Other antidepressants in this class have also shown efficacy, although concern has arisen that some are producing the response by blocking the efficacy of an active tamoxifen metabolite, endoxifen, which utilizes the same CYP2D6 pathway in the liver^22^. The data suggest that citalopram and venlafaxine are least likely to effect CYP2D6 activity and that paroxetine and fluoxetine are more potent inhibitors and should be avoided^35^.

The anticonvulsant gabapentin has been studied for hot flash management. A 2005 study by Pandya *et al.* randomized 420 women with breast cancer and experiencing at least 2 hot flashes in 24 hours to one of three groups: gabapentin 300 mg daily, gabapentin 900 mg daily, or placebo^23^. After 8 weeks, the 300 mg dose group showed a modest 20% reduction in hot flashes, but the 900 mg dose group showed a reduction of approximately 46%. Interestingly, three quarters of the patients in the 900 mg dose group requested and were granted permission to increase their doses beyond the 900 mg, and they achieved a 67% decrease in hot flash severity.

Megestrol is an oral progestogen that has antitumour activity in postmenopausal patients after failure of other endocrine therapies. A crossover study of megestrol versus placebo demonstrated that hot flashes were reduced by 85% with megestrol 20 mg twice daily^36^. However, *in vitro* studies have shown that progestational agents may increase or accelerate breast cancer development or progression, making megestrol a poor choice for management of hot flashes in women. In contrast, it is a very effective treatment for men with prostate cancer who are on hormone manipulation and suffering hot flashes.

In 2007, a case series using oxybutynin for management of hot flashes was reported by members of the breast disease site team at the London Regional Cancer Program. This antimuscarinic agent was observed to reduce hot flashes, providing a complete response in approximately 70% of patients who had been refractory to other treatments. The recommended dose is 2.5 mg either once or twice daily; side effects typical of anticholinergic medications are generally mild and well tolerated^26^.

## 5. SUMMARY

Hot flashes are a significant problem for quality of life in breast cancer patients. They likely contribute to poor compliance with hormone-suppressive therapies. Family physicians, oncologists, and nurses can play an important role both in assessing hot flashes and in reviewing and tailoring treatment options to individual patient needs.

## Figures and Tables

**TABLE I:** Incidence of hot flashes in the major trials of aromatase inhibitors

Reference	Trial short name	Hot flashes (%) with				*p* Value
		Anastrozole	Tamoxifen	Letrozole	Exemestane	
Bonneterre *et al.,* 2001^1^	atac	35.7	40.9			0.0001
Bonneterre *et al.,* 2003^15^	target	19.6	18.8			Not reported
Coombes *et al.,* 2004^3^	ies		39.6		42	0.082
Coates *et al.,* 2007^2^	big 1-98		38	33.5		0.001

**Table II:** Review of treatment options for hot flashes in breast cancer patients

Reference and management option	Effect	Reduction (%) in			Placebo	*p* Value
		Composite hot flash score	Severity	Frequency	(% response)	
Freedman and Woodward, 1992^19^						
Paced respiration	Reduced distress			39		0.02
Freedman *et al.,* 1995^20^						
Paced respiration	Reduced distress			44		0.001
Loprinzi *et al.,* 2000^21^						
Oral clonidine 0.1 mg once daily	Improved quality of life			34.2	21.1	0.001
			11.7		8.5	0.18
		42.6			24.1	0.002
Stearns *et al.,* 2003^22^						
Venlafaxine 75 mg daily	Improved quality of life and libido			46	19	0.001
		61			27	0.001
Pandya *et al.,* 2005^23^						
Gabapentin 300 mg daily	Decreased pain, worse appetite			28	18	0.0002
				41	18	
			33		21	0.0001
Gabapentin 900 mg daily	Decreased pain, worse appetite		49		21	0.0001
Elkins *et al.,* 2008^24^						
Hypnosis	Improved mood and sleep, decreased interference with daily life	68			Not used	0.001
Fenton *et al.,* 2008^25^						
Relaxation training	Reduced distress		50	22 (clinically nonsignificant)	Not used	0.0001
Sexton *et al.,* 2007^26^						
Oxybutynin 2.5 mg twice daily			Improved	Improved		
			(retrospective chart review)			

## Footnotes

a Thurlimann B, for the big 1-98 Collaborative. Letrozole as adjuvant endocrine therapy for postmenopausal women with receptor-positive breast cancer: first results of ibcsg 18-98/big 1-98. Presented at the Ninth International Conference on Primary Therapy of Early Breast Cancer; January 26, 2005; St. Gallen, Switzerland
